# Fabrication of scaffold-free tubular cardiac constructs using a Bio-3D printer

**DOI:** 10.1371/journal.pone.0209162

**Published:** 2018-12-17

**Authors:** Kenichi Arai, Daiki Murata, Ana Raquel Verissimo, Yosuke Mukae, Manabu Itoh, Anna Nakamura, Shigeki Morita, Koichi Nakayama

**Affiliations:** 1 Department of Regenerative Medicine and Biomedical Engineering, Faculty of Medicine, Saga University, Saga, Japan; 2 Department of Thoracic and Cardiovascular Surgery, Faculty of Medicine, Saga University, Saga, Japan; Osaka Shiritsu Daigaku, JAPAN

## Abstract

A major challenge in cardiac tissue engineering is the host’s immune response to artificial materials. To overcome this problem, we established a scaffold-free system for assembling cell constructs using an automated Bio-3D printer. This printer has previously been used to fabricate other three-dimensional (3D) constructs, including liver, blood vessels, and cartilage. In the present study, we tested the function *in vivo* of scaffold-free cardiac tubular construct fabricated using this system. Cardiomyocytes derived from induced pluripotent stem cells (iCells), endothelial cells, and fibroblasts were combined to make the spheroids. Subsequently, tubular cardiac constructs were fabricated by Bio-3D printer placing the spheroids on a needle array. Notably, the spheroid fusion and beat rate in the constructs were observed while still on the needle array. After removal from the needle array, electrical stimulation was used to test responsiveness of the constructs. An increased beat rate was observed during stimulation. Importantly, the constructs returned to their initial beat rate after stimulation was stopped. In addition, histological analysis shows cellular reorganization occurring in the cardiac constructs, which may mimic that observed during organ transplantation. Taken together, our results indicate that these engineered cardiac tubular constructs, which address both the limited supply of donor tissues as well as the immune-induced transplant rejection, has potential to be used for both clinical and drug testing applications. To our knowledge, this is the first time that cardiac tubular constructs have been produced using optimized Bio-3D printing technique and subsequently tested for their use as cardiac pumps.

## 1. Introduction

Heart failure is a leading cause of death worldwide, and organ transplantation has been used as an effective therapeutic method for end-stage heart failure [1–3]. Unfortunately, organ availability remains a serious problem, especially because the number of heart failure patients has steadily increased in recent years. In addition, the detrimental effects of immunosuppression treatments, which are required for successful organ transplantation, are also a concern [4]. Surgical interventions, such as implantation of ventricular assist devices, can be used to circumvent some of the known issues with organ transplantation, but only in a limited number of patients [5]. However, these procedures are associated with other surgery-related risks, such as blood clots, bleeding, infection, and device malfunction [6].

Recent advances in tissue engineering technology have greatly enhance organ transplantation as human tissues and organs for medical applications can now be manufactured *in vitro* using human cells [7]. Indeed, various cell types have been evaluated for their use in tissue engineering, however our attention attract induced pluripotent stem cells (hiPSCs) which can differentiate into functional cardiomyocytes [8–12] and possess features that render them biocompatible, functional, and safe for long-term clinical applications.

To obtain the most desirable three-dimensional (3D) construct, fulfilling all the necessary characteristics of the engineered cardiac tissue, two basic technics scaffold-based and scaffold-free were evaluated.

Scaffold-based cardiac tissue engineering is useful as it provides a foundation for the construction of the 3D environment and can help modulate specific heart functions. Several groups have fabricated cardiac constructs from hydrogel mixtures, such as matrigel and collagen, and rat heart cells [13,14]. Further, when electrical stimulation was applied to these constructs for 5 days, the engineered cardiac tissue displayed an enhanced inotropic reserve. However, this technique has several disadvantages. For example, the materials used to assemble the scaffold-based constructs (e.g., collagen) are immunogenic. It is also difficult to reproduce the native microstructure and mechanical properties of heart *in vitro* with these techniques [15].

Alternatively, scaffold-free tissue engineering has also been investigated with regard to organ transplantation. For example, cell sheets are one of the most advanced methods in the field of tissue engineering [16] and have been used for various clinical applications, including repairing heart damage [17,18]. Sekine *et al* reported the improvement of cardiac function after the transplantation of engineered cardiac cell sheets [19]. Although cell sheets are both effective and versatile, this technology limited the thickness of 3D cardiac construct only up to 200 μm. Other scaffold-free method is using 3D bio-printed materials as a molding template to construct 3D tissues. Disadvantage of this technic is that constructs created using bio-paper, such as agarose gel [20] are difficult to be removed after construct fabrication because the complicated shapes are fabricated using this system.

To overcome these issues, we developed a new Bio-3D printing technology in which cells are aggregated into spheroids that can then be printed onto a needle array according to the desired 3D design. Thanks to totally scaffold-free environment, the fusion of spheroids can be observed at any point. Subsequently, the constructs can be removed from the needle array and matured in a bioreactor. Noguchi *et al* [21], in their study showed fabrication of scaffold-free cardiac patches using cardiac spheroids. Following the *in vivo* application of scaffold-free Bio-3D-printed cardiac patches, Ong *et al* [22] observed increased vascularization and patch engraftment. Although the use of these 3D-printed tissue is becoming more widely accepted in the tissue engineering field, there are still a number of applications that have not been investigated with regard to organ transplantation in the treatment of cardiac failure and damage. Further, Sekine *et al* [23] reported transplantation of pulsatile myocardial tubes fabricated with cell sheets. The transplantation of rat cardiac cell sheets around the abdominal aorta led to change of inner pressure evoked by their contraction. And Seta *et al* [24] reported about the application of fabricated human tubular cardiac tissues derived from iPS cells to the rat inferior vena cava.

In the present study, we fabricated a scaffold-free tubular cardiac construct which is functional as a cardiac pump. Our analysis includes optimization of the conditions for cardiac spheroid assembly as well as electrical stimulation of the printed tubular cardiac constructs. To our knowledge, this is the first time that cardiac tubular constructs have been produced using this Bio-3D printing technique and subsequently tested for their use as cardiac pumps.

## 2. Materials and methods

### 2.1. Cell culture

Human umbilical vein endothelial cells (HUVECs) and normal human dermal fibroblasts (NHDFs) were purchased from Lonza, Inc. (Walkersville, MD, USA) and cultured in EBM-2 and FBM-2. Both cell types were cultured in a humidified 37°C, 5% CO_2_. Human iPSC-derived cardiomyocytes (iCells) were purchased from Cellular Dynamics International, Inc. (CDI, Madison, WI, USA) [25,26]. iCells were thawed and cultured according to the manufacturer’s instructions. Cryopreserved iCell cardiomyocytes were thawed for 4 min in a water bath at 37°C, diluted in iCell plating medium (CDI), and used for spheroid formation.

### 2.2. Cardiac spheroid formation

iCells, HUVECs, and NHDFs were suspended and mixed at several ratios (Table 1) and seeded into ultra-low attachment 96U-well plates (SUMITOMO BAKELITE, Tokyo, Japan) to form cardiac spheroids containing a total of 35,000 cells each. Culture media specific for each cell type were combined in the same proportion as the respective cells for the various mixtures. The spheroids were cultured in a humidified 37°C, 5% CO_2_ incubator. After 2 days, the medium was changed from iCell plating medium to iCell maintenance medium and then changed every 2 days. The cardiac spheroids formed were used for further analyses and fabrication of the cardiac constructs. The size and roundness of cardiac spheroids were measured by Bio-3D printer software. The formula used to calculate roundness of cardiac spheroids was as follows. Circle rate (%) = 100-(R-r)/R×100. And “R” is radius of minimum circumscribed circle, “r” is radius of inscribed circle.

**Table 1:** Percentage of each cell type used to form cardiac spheroids in this study.

	A)	B)	C)	D)	E)
**iCell**	**100%**	**80%**	**70%**	**60%**	**50%**
**HUVEC**	**0%**	**10%**	**20%**	**20%**	**25%**
**NHDF**	**0%**	**10%**	**10%**	**20%**	**25%**

### 2.3. Fabrication of scaffold-free cardiac constructs using a Bio-3D printer

**Fig 1:**
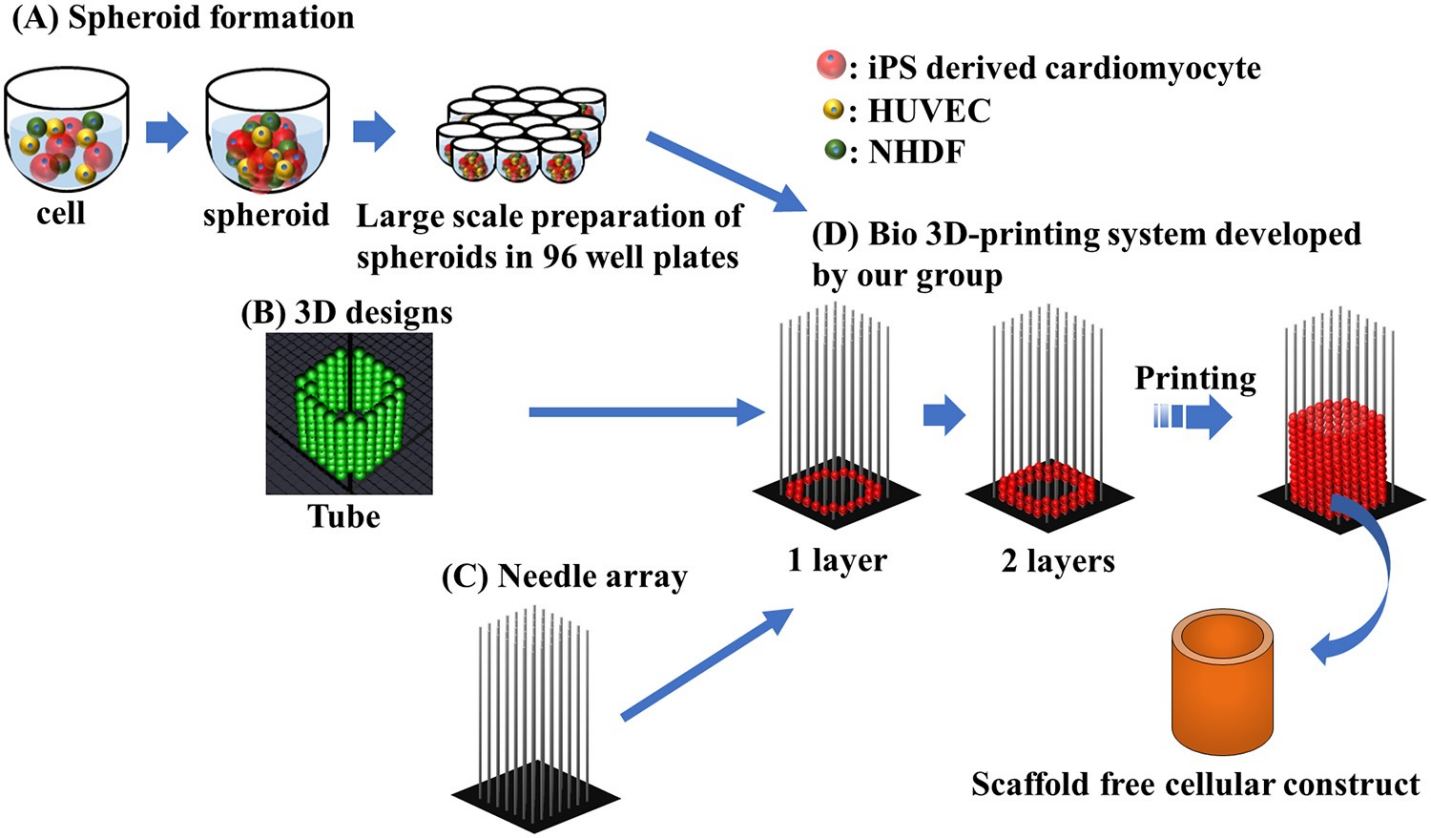
The Bio-3D printing process. Cells aggregate as spheroids (A). The desired 3D design is composed using the needle array software (B) and the appropriate needle array (C) is selected and prepared. The spheroids are then printed onto the needle array (D).

A Bio-3D printer (Cyfuse Biomedical K.K., Tokyo, Japan) was used to assemble the cardiac spheroids into scaffold-free cardiac constructs. The method used to fabricate these constructs has been previously reported [27] and a diagram is shown in Fig 1, which includes the 3D designs for a tubular structure (Fig 1B). Constructs were fabricated using 7-day-old cardiac spheroids (Fig 1A) and cultured on the needle arrays for an additional 7 days (Fig 1D). After removal from the needle array, the cardiac tubular constructs were cultured on 22-gauge plastic catheters (Terumo, Tokyo, Japan) in a bioreactor for 7 days, followed by fixation and analysis.

### 2.4. Histological and immunohistochemical analysis

Cardiac spheroids and constructs were fixed in 10% Formalin Neutral Buffer Solution (Wako Pure Chemical Industries, Ltd, Osaka, Japan) for 48 h at 4°C. After fixation, the samples were washed three times with phosphate-buffered saline (PBS), embedded in paraffin, and cut into 5 μm sections. These sections were then stained with hematoxylin and eosin and used for immunostaining. Primary antibodies against troponin T (dilution 1:100; MS-295-P0, Thermo Fisher Scientific, Inc., Massachusetts, USA), CD31 (dilution 1:75; NCL-CD31-1A10, Leica Biosystems, Wetzlar, Germany), and CD90 (dilution 1:100; ab92574, Abcam Plc., Cambridge, UK) were used. To confirm cell viability, TUNEL assay was performed with in situ cell death detection kit (Roche Applied Science, Penzberg, Germany) according to the manufacturer’s instructions. After deparaffinization and hydration via an ethanol gradient, the sections were treated with a 0.3% (v/v) H_2_O_2_ solution for 10 min. Then, they were washed two times with tris-buffered saline (TBS) and incubated with primary antibody diluted in blocking solution at 4 °C overnight. After washing twice with TBS, the samples were incubated with secondary antibody at room temperature for 30 min, washed two times with TBS, and treated with DAB (Agilent Technologies Japan Ltd, Tokyo, Japan) solution. After mounting, the sections were observed and imaged using a BZ-X700 microscope (Keyence, Osaka, Japan).

### 2.5. Contraction analysis

**Fig 2:**
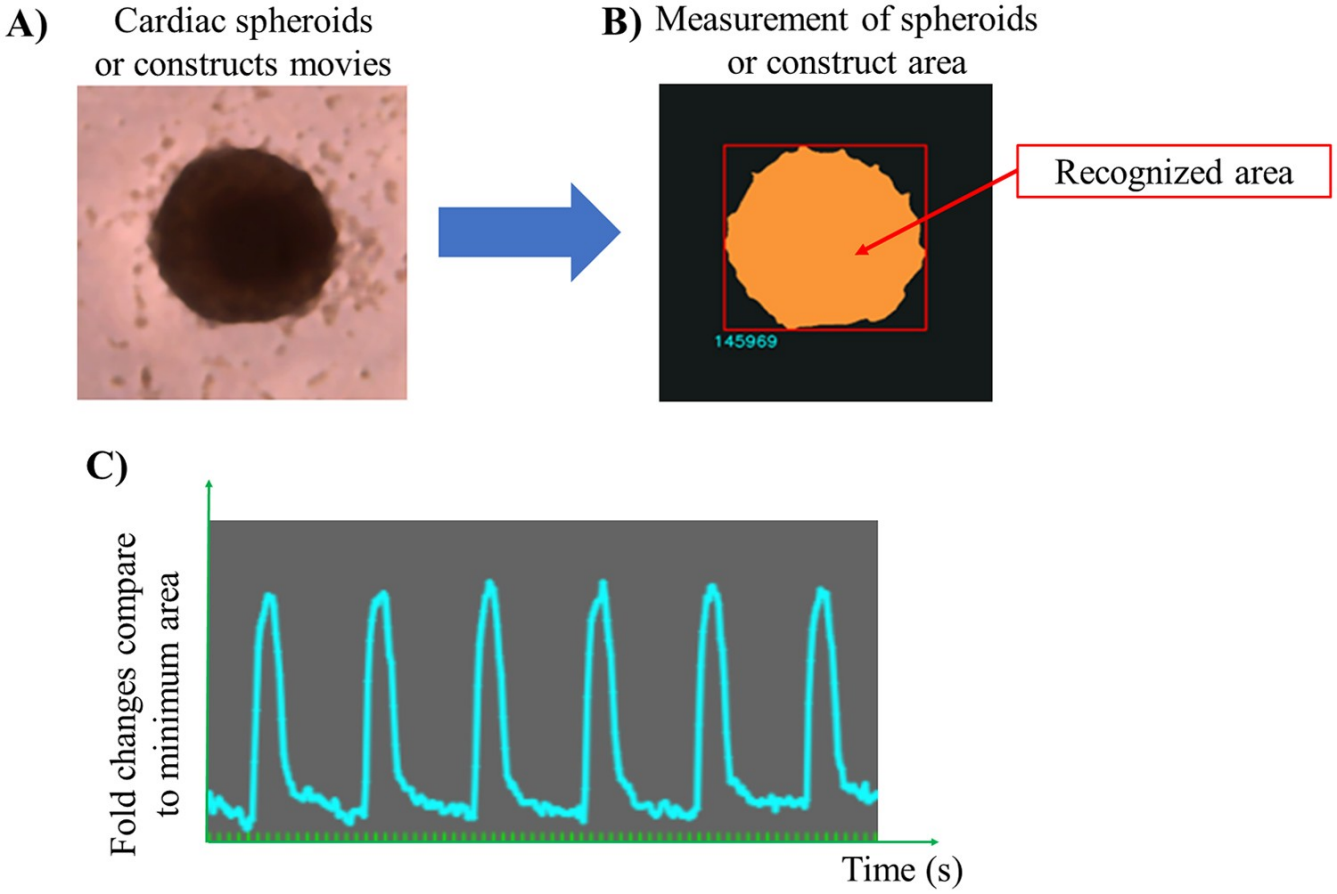
Motion analysis of the contractile area in cardiac spheroids and constructs. The magnitude of each contraction in the cardiac spheroids and constructs was recorded and individual frames were isolated (A). The spheroid’s and construct’s area were then measured in each frame using our in-house software (B). The fractional area changes in the spheroids and constructs were calculated and graphed (C).

We developed an in-house analysis software that can recognize and measure changes in cardiac spheroid area using video recordings (Fig 2). This software was also used to analyze the beat rate and contractile activity of the constructs. Each frame in the recorded movies was converted to binary and used to calculate the contracted area (Fig 2A and 2B). The fractional area changes were calculated as the contracted area/minimum area (Fig 2C). The videos were recorded using a digital camera (Leica MC120 HD, Leica Biosystems, Wetzlar, Germany) mounted on an inverted bright field (Leica DMi1, Leica Biosystems, Wetzlar, Germany) or stereoscopic microscope SZX7 (Olympus, Tokyo, Japan). Cardiac spheroids were recorded and analyzed after 10 days of culture.

### 2.6. Electrical stimulation

**Fig 3:**
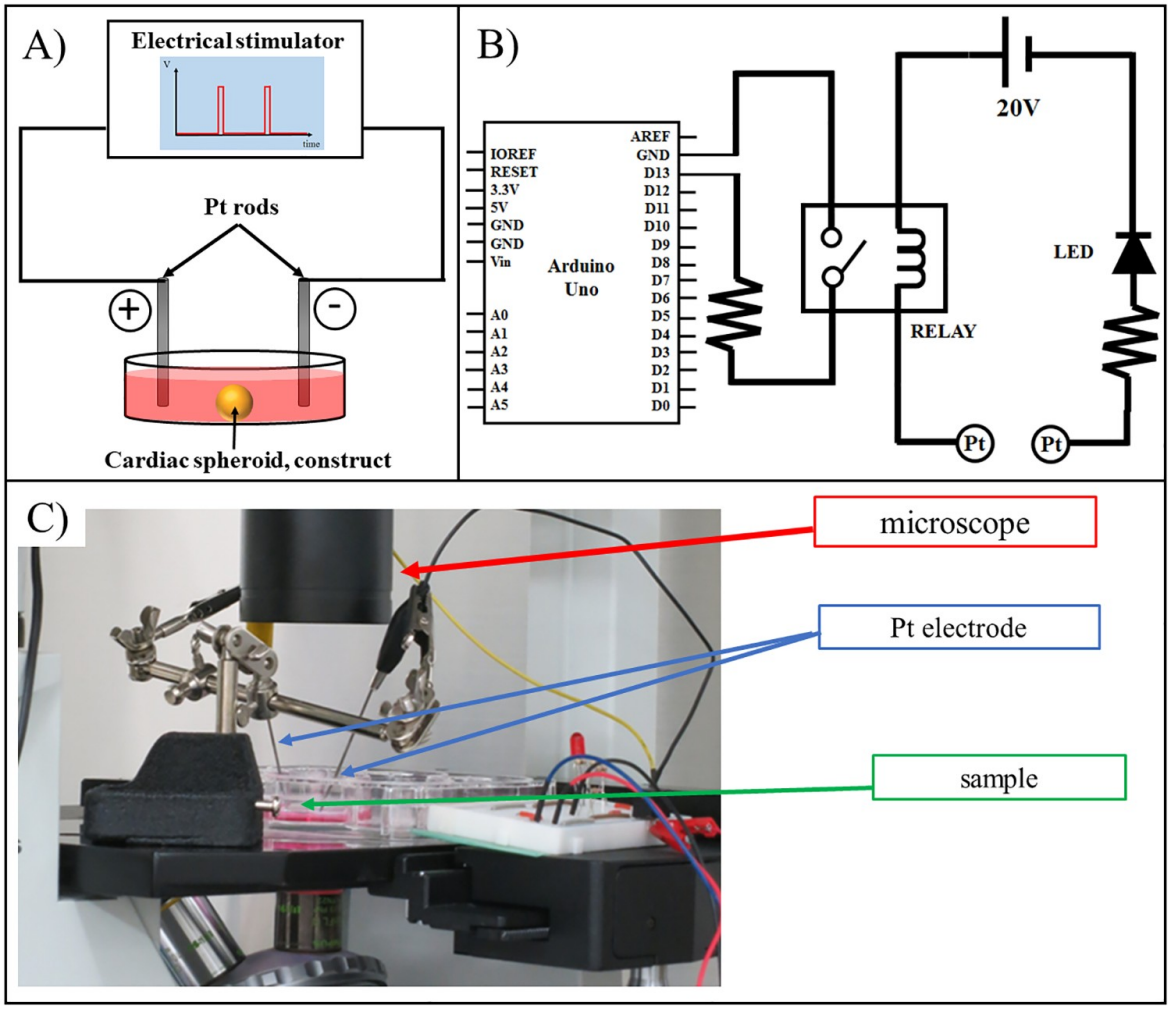
Electrical stimulation system. Basic diagrams of the electrical stimulation system (A) and the electric circuit (B). The electrical stimulator generates pulses which are then transmitted to the medium in the dish. To image the constructs, the samples were placed between the Pt electrodes and the entire system was set on a microscope (C).

After 14 days of culture (printing day is set as day 0), the cardiac constructs were transferred to a chamber with two platinum rods connected to an electrical stimulation device (Fig 3A). Fig 3B shows the electronic circuit of this system. To stimulate the cardiac construct, a PSW 80–13.5 was used as the electric power supply (Good Will Instrument Co., Ltd, New Taipei City, Taiwan) in conjunction with the computer program Arduino Uno. A diagram of the electrical set up is shown in Fig 3C. Constructs were stimulated with bipolar electrical pulses of 20 V and 2 Hz that lasted for 10 ms and were repeated every 490 ms. After stimulation, the data were analyzed using an analysis software developed in-house.

## 3. Results

### 3.1. Characterization of cardiac spheroids containing iCells, endothelial cells, and fibroblasts

**Fig 4:**
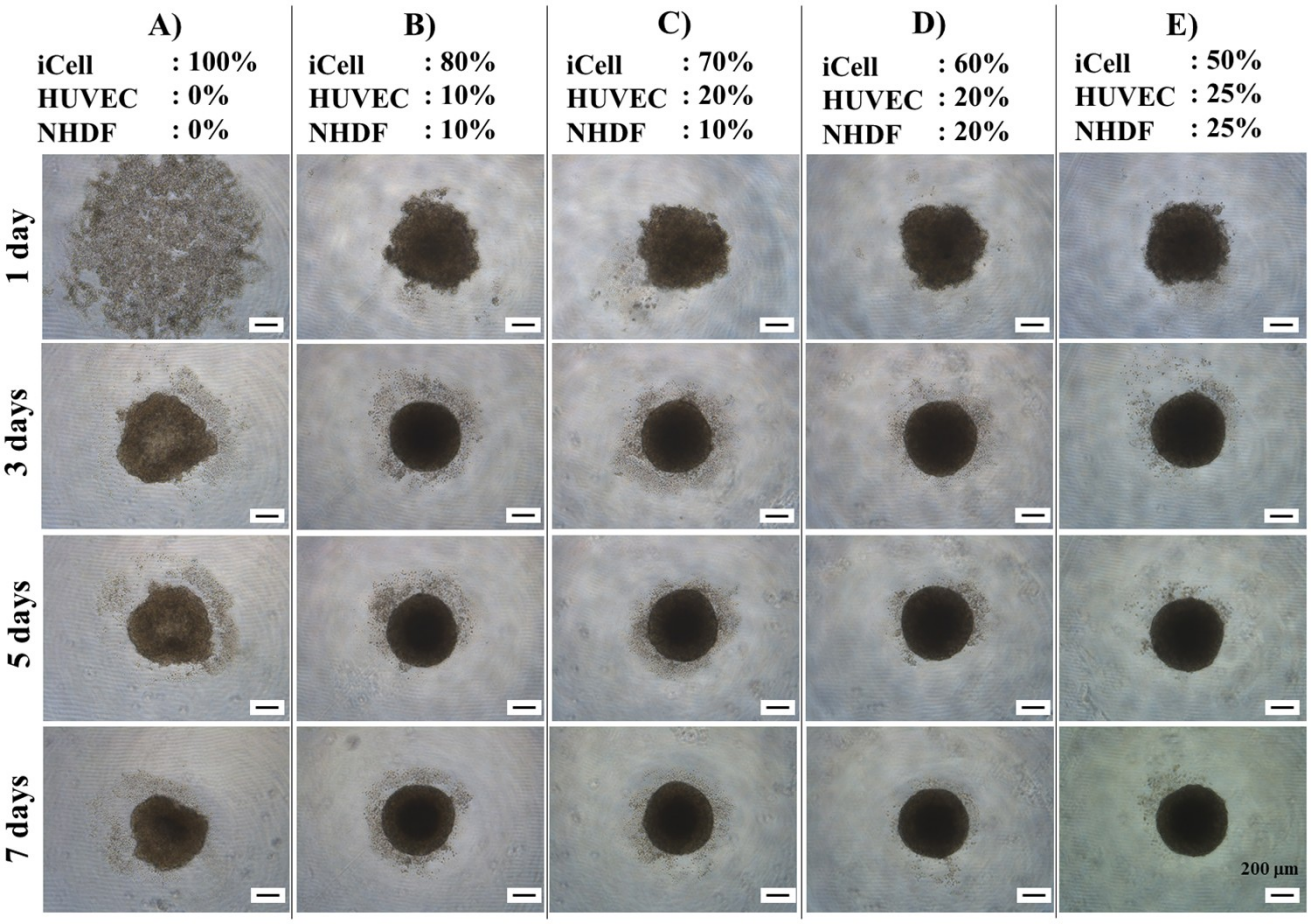
Time-lapse imaging of cardiac spheroid formation. Cardiac spheroids containing different percentages of iCells, HUVECs, and NHDFs were prepared and their formation process was imaged and recorded on days 1, 3, 5, and 7.

**Fig 5:**
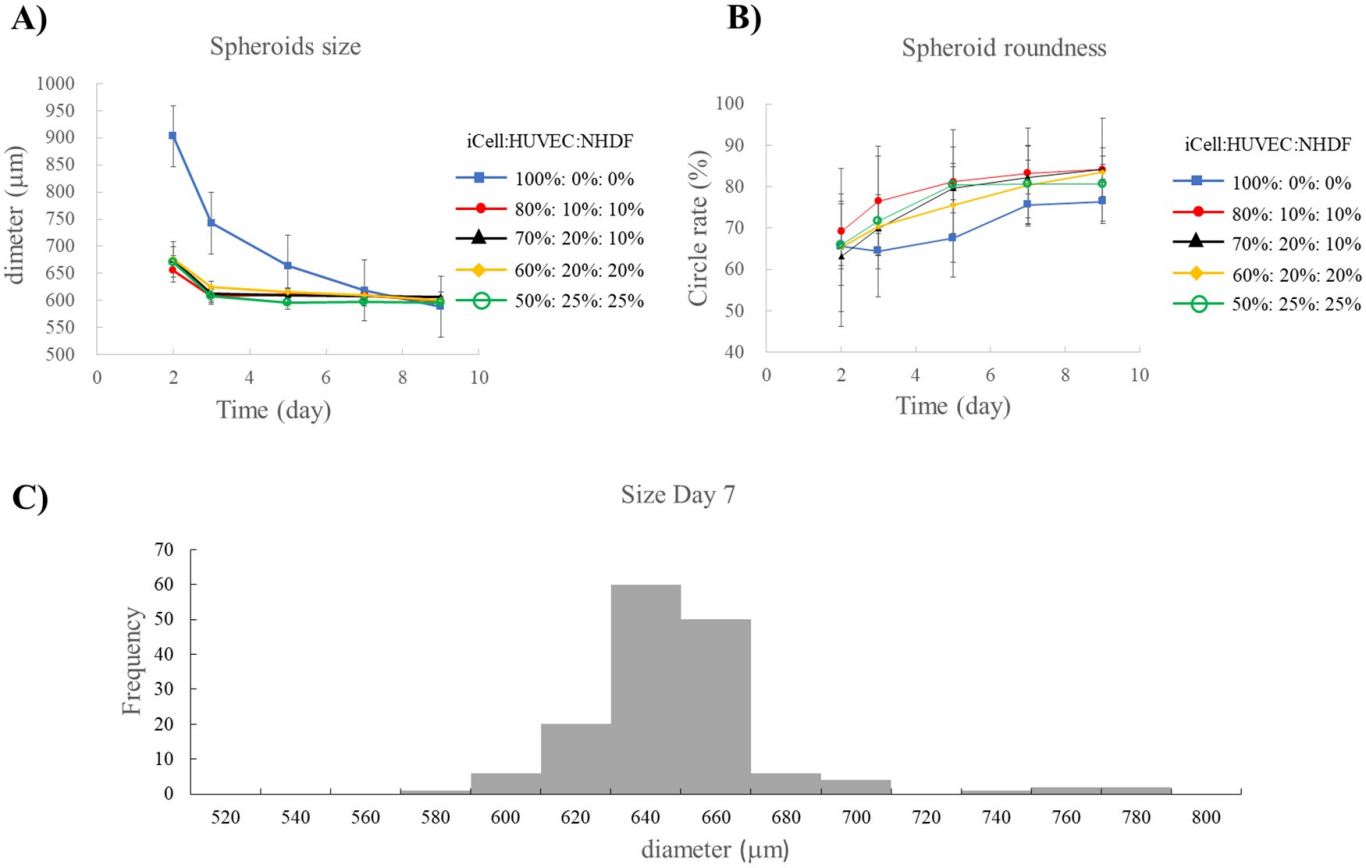
Spheroid size and roundness change over time. Changes in size (A) and roundness (B) were measured in the cardiac spheroids composed of the specified cell mixtures for 9 days. The size of all cardiac spheroids was measured at day 7, before being used to fabricate the cardiac constructs (C).

Cells were seeded on ultra-low attachment plates and cardiac spheroid formation was observed at days 1, 2, 5, and 7 (Fig 4). After 3 days of culture, the various mixtures of cells began to aggregate to form cardiac spheroids that had stable roundness in 5 days. Notably, suspensions containing 100% iCells only formed spheroids after 7 days of culture. In addition, the size and roundness of the mixed cell spheroids with all three cell types showed greater uniformity at day 7, approximately 600 μm and 80%, compared to spheroids composed only of iCells (Fig 5). These data indicate that the addition of fibroblasts and endothelial cells promotes rapid cell self-organization and enhances cardiac spheroid stability with regards to shape and size.

### 3.2. Contractile activity of cardiac spheroids

**Fig 6:**
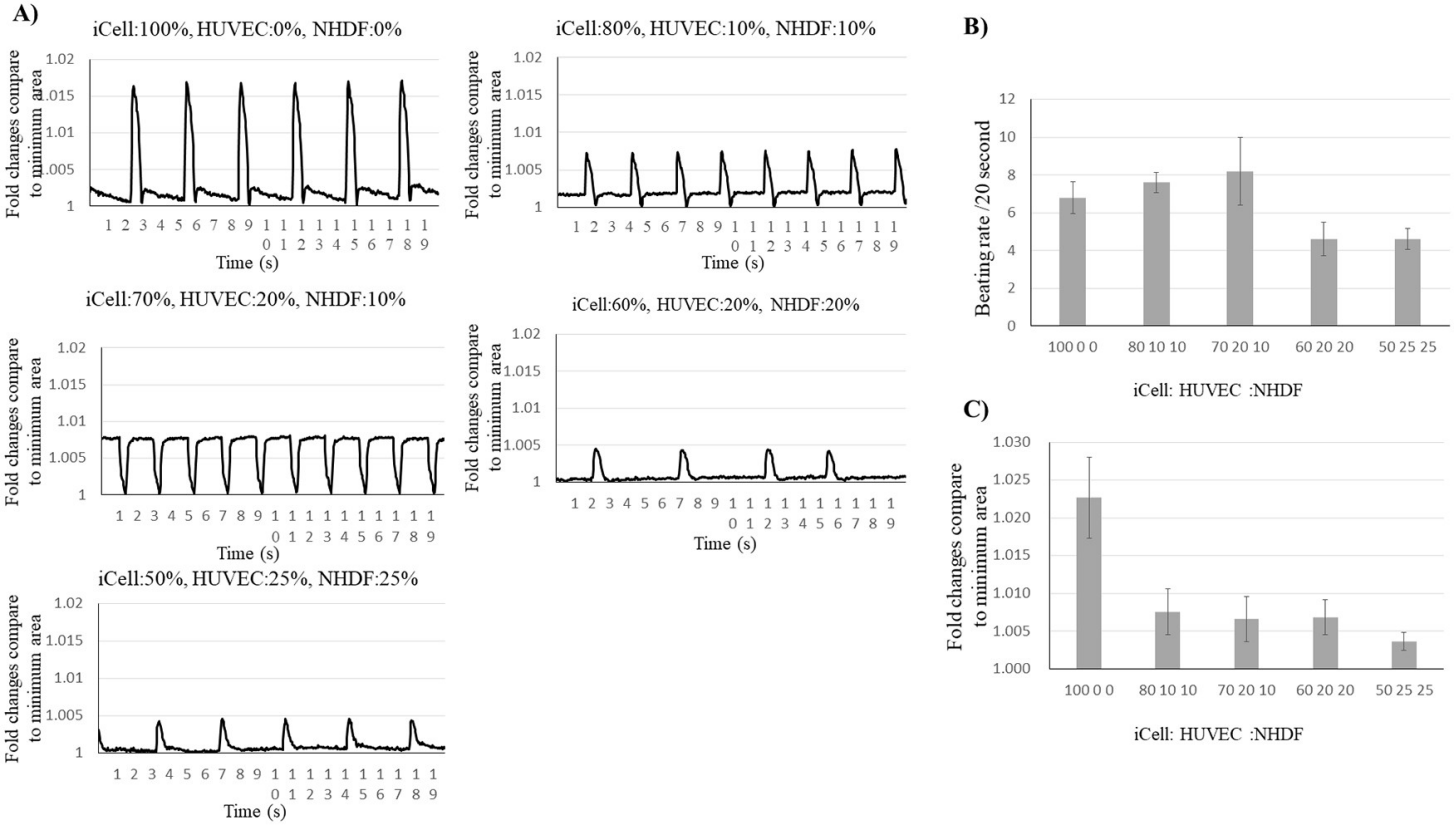
Motion analysis of the contractile cardiac spheroids. The contraction in five types of spheroids were recorded (A), and the beat rate (B) and contraction (C) were analyzed.

After 10 days of culture, cardiac spheroids were recorded individually and analyzed with software developed in-house. Spheroids were prepared using varying percentages of iCells (100%, 70%, 60%, and 50%). Contraction and beat rate were examined. Interestingly, the number of cardiomyocytes in the spheroids appeared to be correlated with a wider beat amplitude (Fig 6).

### 3.3. Histological analysis of cardiac spheroids

**Fig 7:**
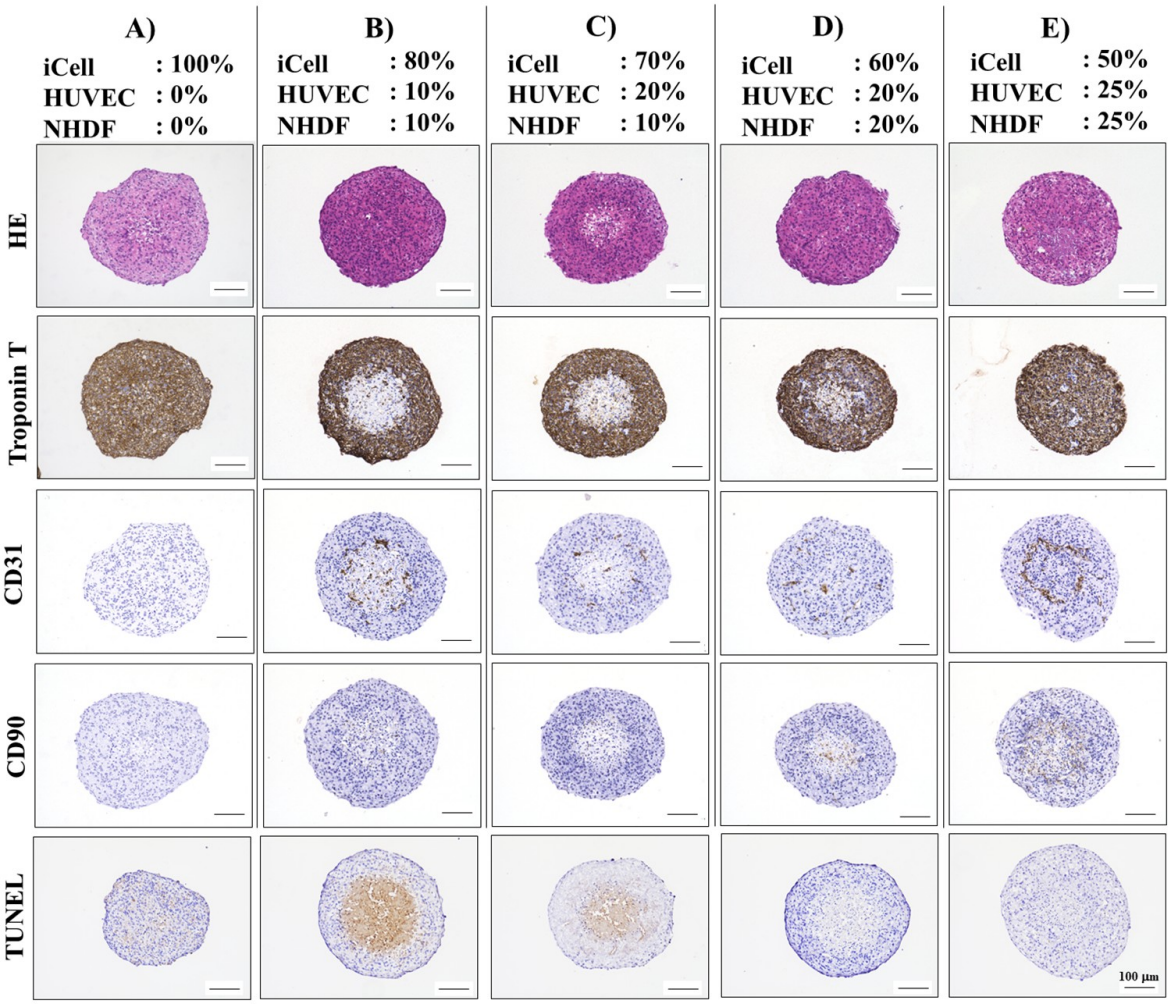
Histological and immunohistochemical analysis of five types of cardiac spheroids. Cardiac spheroids containing different percentages of iCells, HUVECs, and NHDFs were fixed and stained after 7 days. Troponin T is a marker for iCells, while CD31 and CD90 are markers for HUVECs and NHDFs, respectively.

Spheroid formation and cell spatial distribution within the spheroids analyzed histologically using hematoxylin and eosin staining (Fig 7). All cardiac spheroids were maintained at high cell densities. Notably, although troponin T expression in the spheroids fabricated from the 80:10:10 mixture of iCells/HUVECs/NHDFs was observed at the outer rim (Fig 7B), spheroids formed from the 50:25:25 mixture had more generalized and homogenous expression pattern (Fig 7E). The latter also created the endothelial cells aggregation visualized by the CD31 expression not observed for the others cell ratios (Fig 7B and 7D). Also, TUNEL stain-positive cells were uncommon in the spheroids formed from the 50:25:25 mixture (Fig 7E).

### 3.4. Fabrication of cardiac constructs using a Bio-3D printer

**Fig 8:**
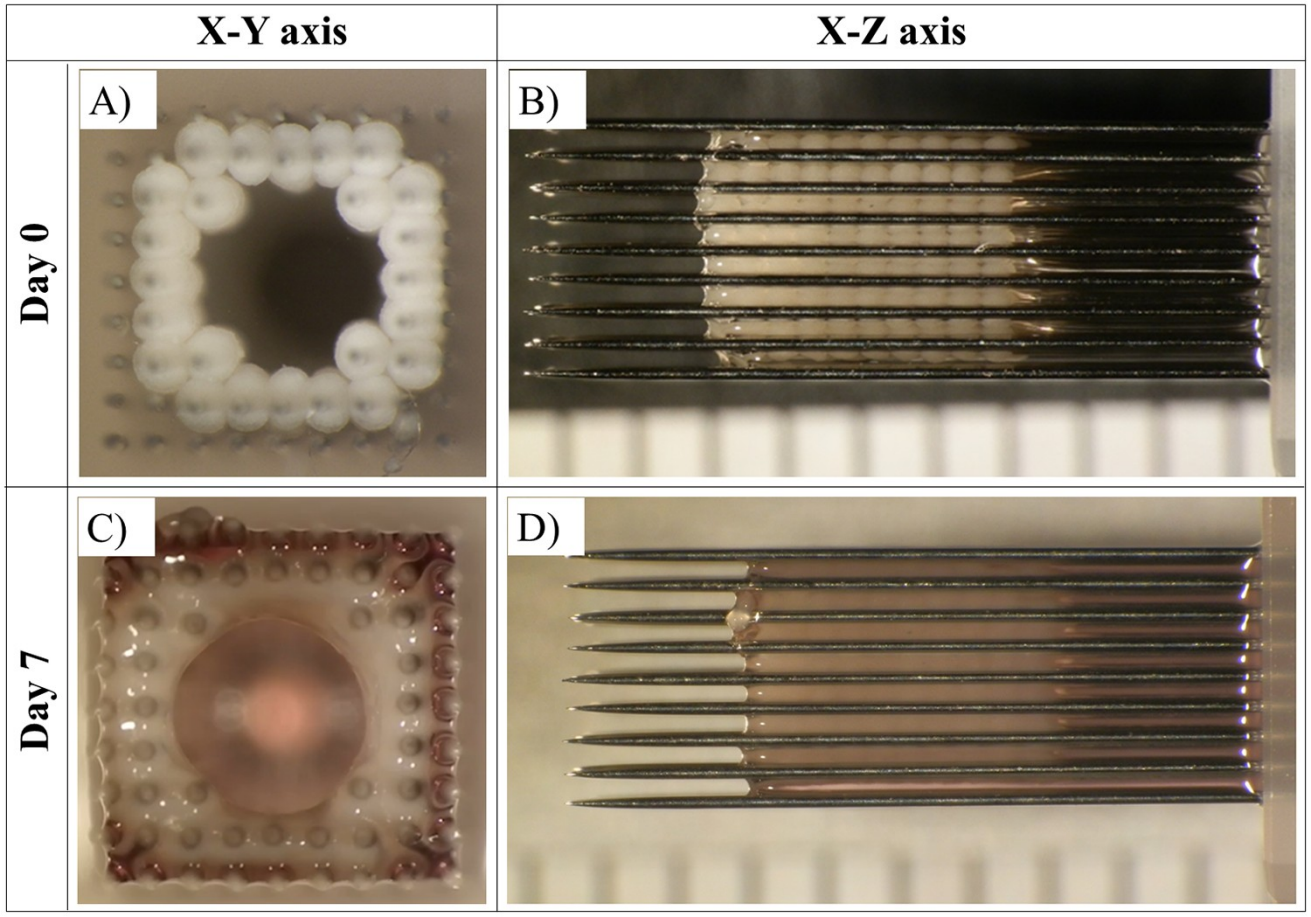
Culture of tubular cardiac constructs on a needle array. Representative images of the fabricated tubular cardiac constructs just after printing (A, B) and after being cultured on the needle array for 7 days (C, D).

**Fig 9:**
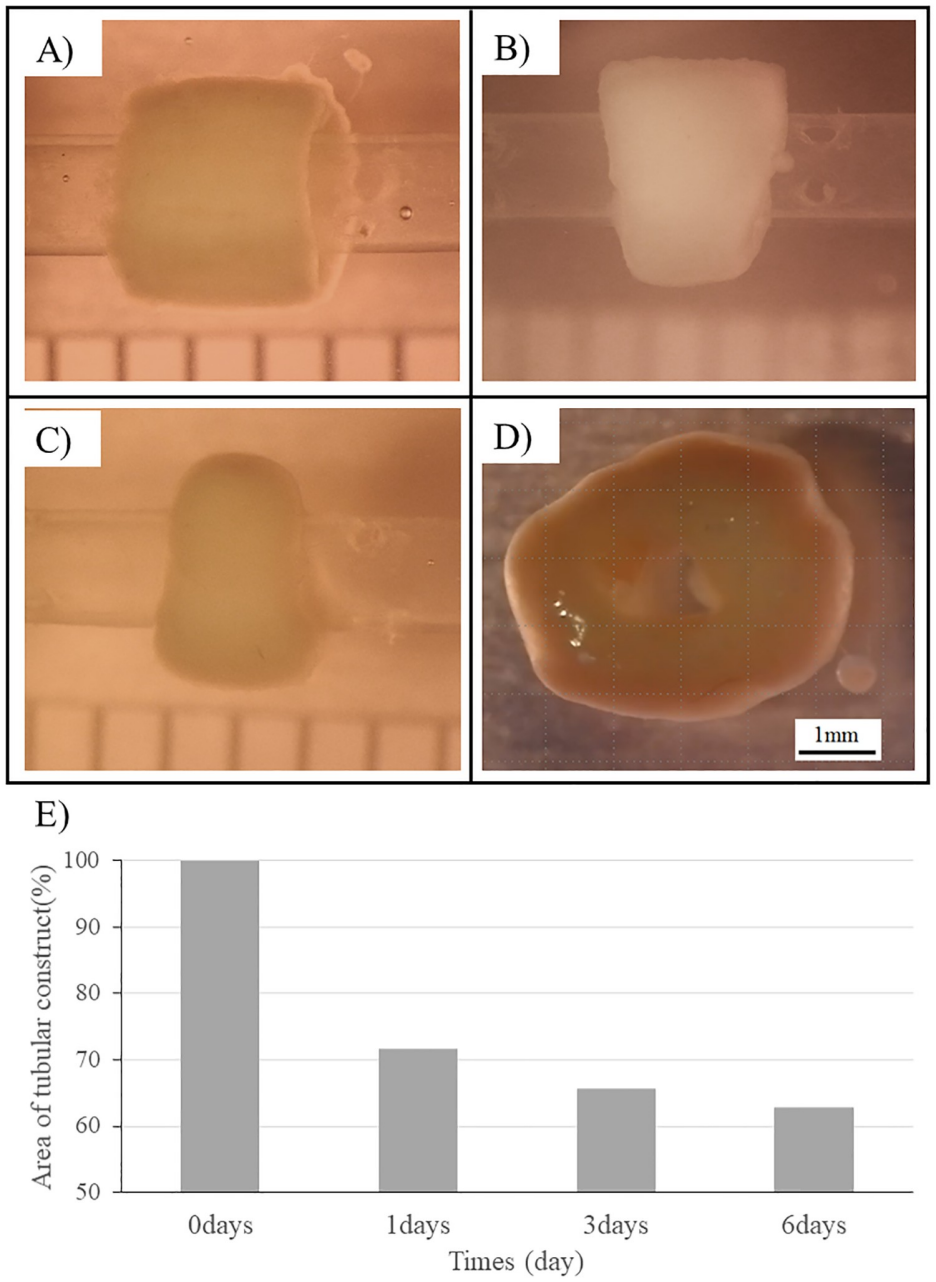
Culture and maturation of tubular cardiac constructs on the plastic catheters. Representative images of the tubular constructs immediately (A), 1 day (B), and 6 days (C) after removal from the needle array as well as after removal from the plastic catheters (D). The areas of the tubular construct were examined (E).

Cardiac spheroids fabricated with the 50:25:25 mixture of iCells, HUVECs, and NHDFs were isolated after 7 days of culture and used to fabricate cardiac constructs via the Bio-3D printer (Fig 8A and 8B). The cardiac constructs were cultured on the needle arrays for an additional 7 days (Fig 8C and 8D). After 1 day, spheroid fusion and contraction were observed under the microscope while still on the needle array (S1 Movie). After removal from the needle array on day 7 post-printing, the constructs were cultured on plastic catheters for another 7 days (Fig 9A). We then monitored the constructs daily and confirmed that their area gradually decreased over time until it reached approximately 70% compared to their size immediately after removal from the needle array (Fig 9E). Notably, the area of the constructs reached 60% of their original size at day 6 (Fig 9C and 9E).

### 3.5. Electrical stimulation

**Fig 10:**
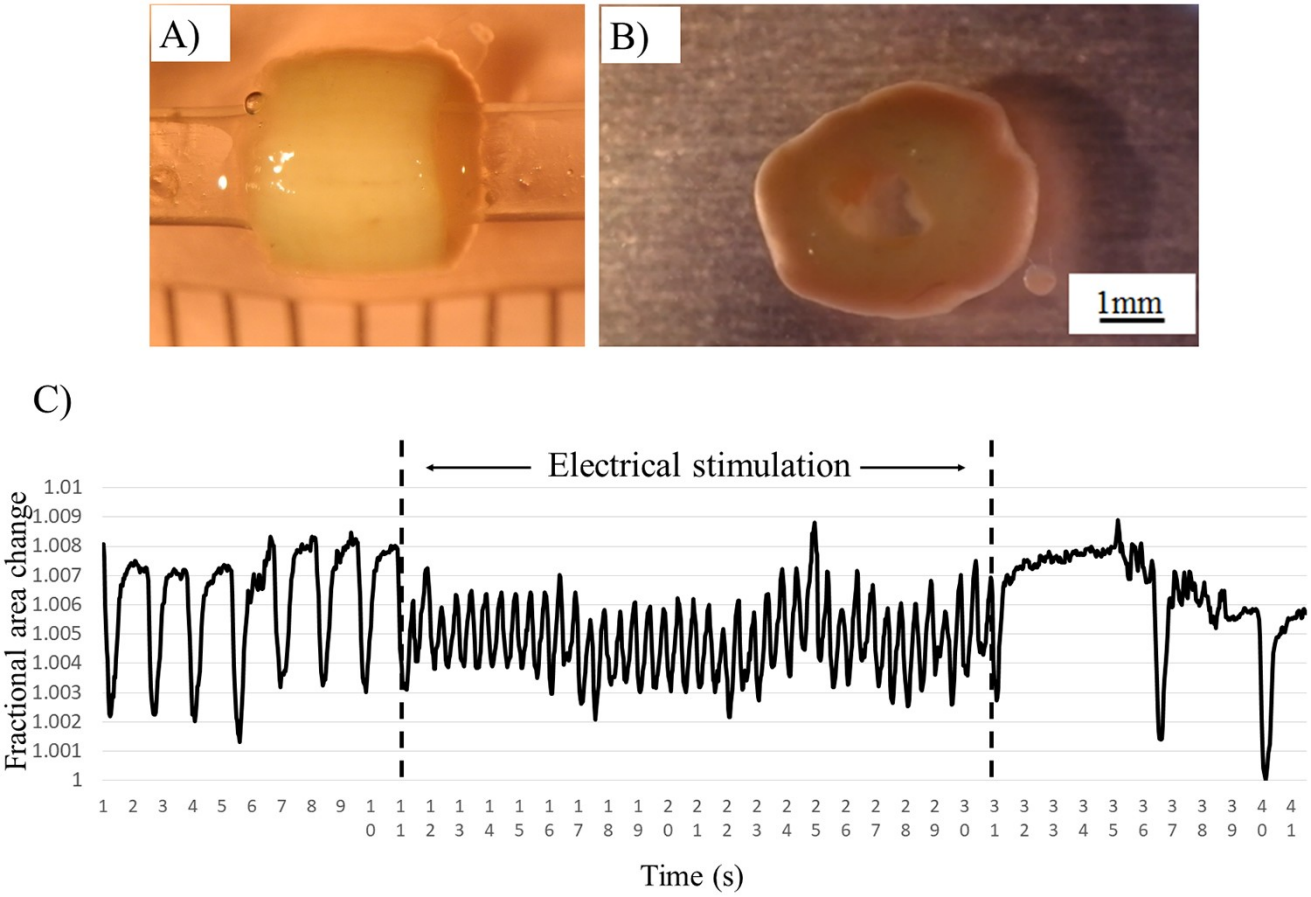
Analysis of the changes in beat rate in response to electrical stimulation of the tubular cardiac constructs. The tubular cardiac constructs were removed from the needle array (A) and cultured for 7 days (B). Electrical stimulation was then applied and its effects were measured and analyzed (C).

To modulate the electrical qualities of cardiac tissues in the fabricated constructs, electrical stimulation was applied to induce beating and contraction. Prior to stimulation, 7 beats per 10 seconds were recorded (Fig 10C). During electrical stimulation, the beat rate increased to 20 beats per 10 second. This is highlighted in S2 Movie. Upon cessation of stimulation, the beat rate in the cardiac constructs temporarily decreased to 2 beats per 10 seconds before returning to its original rate.

### 3.6. Histological examination of cardiac spheroids and constructs

**Fig 11:**
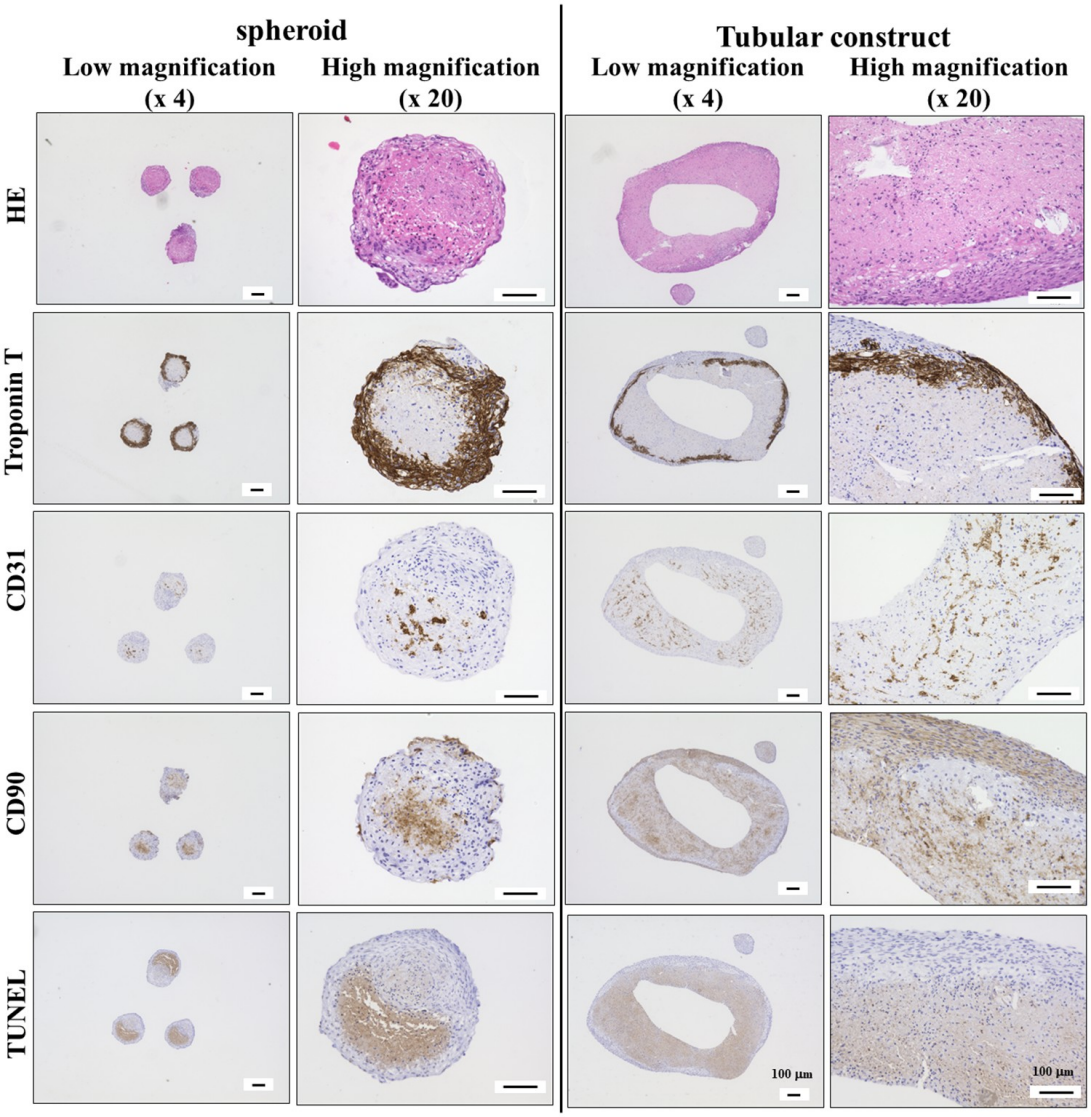
Immunohistochemical analysis of the cardiac constructs. Representative images of spheroids and tubular constructs fabricated with iCells, HUVECs, and NHDFs for 21 days are shown. These samples were observed at low (4×) and high (20×) magnification. iCells were identified with troponin T staining, while HUVECs and NHDFs were stained with CD31 and CD90, respectively.

Cardiac spheroids and constructs were fixed 21 days after spheroid formation, embedded in paraffin, sectioned, and stained for immunohistochemical analysis. Hematoxylin and eosin staining highlighted differences in the overall structure of the spheroids and constructs as well as in the distribution of nuclei (Fig 11). The inner region of the cardiac construct was observed to have low cell density compared with the outer region, and spheroid fusion was observed. Troponin T expression, a marker of iCells, was observed at the outer surface of the constructs, whereas CD31 expression, an endothelial marker, was mainly present in the inner region. CD31 expression also indicates microvascular-like formation in the construct. In contrast, CD90 expression, a fibroblast marker, was found to be ubiquitous. A small number of positive nuclei indicating apoptotic cells were detected in the inner region of the cardiac construct.

## 4. Discussion

Recent advances in tissue engineering have greatly enhanced transplantation methods used for various organ systems, including the heart. Scaffold-free constructs are the preferred type as they eliminate issues with scaffold-related immunogenicity. Our group recently developed a Bio-3D printer that can be used to fabricate various tissue types, such as liver tissue and blood vessels [27,28]. In the present study, we established a method to manufacture scaffold-free tubular cardiac constructs to be used as functional cardiac pumps in clinical applications.

First, it was necessary to optimize the conditions for cardiac spheroid assembly. To this end, we analyzed various parameters, including spheroid size, roundness, culture time, total cell numbers, and cells composition/ratio to obtain the most optimal spheroids for the construct’s printing. Beauchamp *et al* [29] previously reported iCells have the potential to form beating 3D cell aggregates. Further, Stevens *et al* [30] and the others [21,27] demonstrated that aggregated cardiac patches composed of human embryonic stem cell (ESC)-derived cardiomyocytes, endothelial cells, and fibroblasts developed vascular structures which are crucial for *in vivo* successful transplantation. Our previous study also showed that the addition of fibroblasts promotes rapid cell self-organization into spheroids and spheroids fusion with each other on the needle array [21, 27]. Thus, we used a combination of iCells, endothelial cells, and fibroblasts to form desired cardiac spheroids. 50:25:25 mixture composition of iCells/HUVECs/NHDFs displayed the most optimal results for spheroid’ viability, cardiac functions, kinetic of spheroid formation as well as the endothelial cell aggregation compared to the other cell mixtures. Although the number of cardiomyocytes in cardiac spheroids was directly proportional to their beat rate which means that 100:0:0 mixture composition of cells showed the most synchronous contraction, spheroids formation and following construct maturation were not possible. Also, because Ong *et al* have demonstrated that the cell viability in printed cardiac patches was higher than 90% by TUNEL staining and optical electrical mapping [22], we confirmed the cell viability by the same assay in our cardiac spheroids. Although dead cells in spheroids fabricated from 80:10:10 and 70:20:10 mixture of iCells/HUVECs/NHDFs were observed at the inner parts, in 60:20:20 and 50:25:25 mixture conditions were difficult to detect. Because of the highest viability and potential for functionality and stabilized integrity to mature on the needle array, spheroids from 50:25:25 mixture of iCells/HUVECs/NHDFs were used for further construct fabrication.

To fabricate the cardiac constructs, we used a Bio-3D printer. In this system, the shape of the construct can be controlled while it is loaded and cultured on the needle array. However constructs started to shrink after removed from needle array and cultured on plastic catheter, and its thickness increased. One important characteristic of the constructs is that they must maintain their shape until clinical application. This is difficult not only in our system but also in other systems using hydrogel structure, unless anchors are used [31, 32]. In our system, the culture method after removal from the needle array may need to be improved. The observed increase in construct thickness may cause changes in the oxygen and nutrient supply following cells necrosis [33]. Secondly, although the presence of fibroblasts in spheroids promotes spheroids fusion on the needle array by production of collagen inside constructs. On the other hand, it seems possible that the proliferating fibroblasts inside spheroid and tubular construct during culture *in vitro* may cause fibrosis. In future work, there may be necessary to regulate and control fibroblasts proliferation in tubular cardiac constructs. Although these methods may be enhanced further in future studies, our constructs are still superior to the majority of other scaffold-free 3D cardiac fabrications.

Histological analysis of the cardiac spheroids in the tubular constructs after 21 days of culture was also performed to better understand how the composition of the constructs could affect the function. Rearrangement of the cardiomyocytes in the constructs was observed during cultivation. Troponin T, a marker of iCells, is located at the outer surface of the cardiac constructs, whereas in the 7-day-old spheroids its distribution was found to be ubiquitous. Uchida *et al* reported that the villin protein in human gut organoids from pluripotent stem cells located to the apical surface of epithelium *in vitro* [34]. Although organs receive nutrition and oxygen from blood vessels *in vivo*, fabricated 3D organoids and constructs contact with nutrition and oxygen from their surface. We assume that cardiomyocytes located in the outer surface of the construct because of their great need for a lot of nutrition and oxygen.

Indeed, CD31 expression, which identifies HUVECs, was localized in the inner region of the tubular cardiac constructs. Microvascular-like formation was observed at various regions, supporting our earlier observations. Cellular reorganization in the tubular construct is similar to the phenomenon was observed for endothelial cells, fibroblasts, and smooth muscle cells in constructs implanted in nude rats [27]. This reorganization is not likely to negatively alter construct function and may even be necessary for complete acceptance of the engineered tissue into the host.

To test the ability of our tubular cardiac constructs to participate in active contraction, electrical stimulation was applied. During resting conditions, the beat rate in the human heart ranges from 60 to 70 beats per minute [35]. The beat rate in our constructs was slower, with only 7 beats per 10 seconds (calculated to be approximately 42 beats per minute). However, when electrical stimulation was temporary applied to the tubular cardiac constructs, the beat rate increased to 20 beats per 10 seconds (extrapolated to 120 beats per minute). These results are supported by a previous study showing that the beat rate in scaffold-based cardiac constructs was also increased after application of electrical stimulation for 2 weeks [36]. Thus, our cardiac constructs appear to have the potential to control the beat rate and tissue contraction in clinical applications.

It is important to note that we are not the first to develop functional 3D cardiac constructs for clinical application. Ong *et al* [22] also used a Bio-3D printer to fabricate scaffold-free cardiac patches that were subsequently implanted onto the hearts of nude rats. Their results indicate that patch implantation promoted vascularization and engraftment *in vivo*. The ultimate goal of these cardiac patches is to improve heart function and regeneration of myocardial tissues after injury. However, these cardiac patches have several notable limitations, such as slow conduction velocity and weak mechanical properties which are largely related to the short-term culture followed after printing. To overcome these issues in the present study, the tubular cardiac constructs were cultured onto tubular needle arrays in a bioreactor for 7 days as well as an addition 7 days after removal from the needle array. This culture protocol seems to promote the production of extracellular matrix and fusion of spheroids. Our constructs may have a potential to increase the strength by using bioreactor. To maintain cardiac function and cell viability inside thick cardiac construct is very important. Although dead cells in spheroids fabricated from 50:25:25 mixture of iCells/HUVECs/NHDFs after 7 days were almost not observed, those in inner parts of cardiac tubular constructs after 21 days of culture were confirmed. However, positive signal of TUNEL staining in cardiac construct was weaker, compared to that in cardiac spheroids. This data indicates that the organization of spheroids using Bio-3D printer can improve viability of the cells inside spheroids. And we reported that liver-like constructs predesigned and printed with microchannel structure to enhance medium perfusion during maturation process were improved in the cell viability and the albumin production by supplying medium into the construct [28]. This result indicated that the supply of nutrients and oxygen were important for the fabrication of 3D tissue. In our future work, our cardiac construct predesigned and printed with microchannels are expected to improve cell viability inside thick cardiac constructs.

Our constructs also appear to be superior to cell sheets. Sekine *et al* [19] reported the fabrication method and transplantation of pulsatile myocardial tubes fabricated using cell sheets. These prepared cardiac sheets were wrapped around rat thoracic aorta tissue *in vitro* and implanted into the rat abdominal aorta. Although their implanted cardiac tubes were able to produce independent cardiac pressure, apart from host heart beats, their use requires donor thoracic aorta tissue. Therefore, Seta *et al* reported that the cardiac tubular tissues might be exposed to the ischemic condition for a while after transplantation, because the microvascular formation was not sufficient in the cardiac tubular tissues. Our fabricated constructs can be implanted directly into the abdominal aorta, reducing the dependence on donor tissues.

The potential uses of our scaffold-free cardiac tubular constructs are numerous and include both clinical (e.g., transplantation) and *in vitro* (e.g., drug testing) applications. In fact, several research groups have used cardiac constructs and monolayer cardiac cultures *in vitro* to investigate drug responsiveness [29,37]. Beauchamp *et al* [29], for example, evaluated cardiotoxicity by testing various drugs on iCell spheroids *in vitro*, using the observed contractile changes of the spheroids to effectively represent cardiomyocyte toxicity. The tubular cardiac constructs fabricated in the present study using iCell spheroids could similarly be used to evaluate drug-induced contractile changes and cytotoxicity. Although additional research is required to establish their full potential, our tubular cardiac constructs could be used in several ways to enhance our understanding of cardiac function and transplantation.

In conclusion, we fabricated scaffold-free cardiac tubular constructs using a Bio-3D printer that, in our preliminary study, appear to be fully functional without the addition of donor tissue. Notably, contraction, spheroid fusion, and cellular reorganization were observed, all of which occur in a manner that is similar to donor tissues. Future studies using these constructs will focus on *in vitro* (such as calcium transport and drug response) and *in vivo* (such as contractile force and biocompatibility) functions and applications. To our knowledge, this is the first time that cardiac tubular constructs have been produced using this Bio-3D printing technique and subsequently tested for their use as cardiac pumps. This study addresses major issues in organ transplantation which involve the limited supply of donor tissues as well as immune-induced transplant rejection. These are overcome by eliminating the need for donor tissue and limiting the amount of foreign material in the construct (i.e., scaffold, bio-paper, etc.) that could cause an immune response in the host. We provide a novel optimized protocol for cardiac construct fabrication that can be used to prepare tissues for both drug testing and clinical applications.

## Supporting information

S1 Movie Tubular cardiac constructs after removal from the needle array. This movie shows the contraction of tubular cardiac constructs before and just after removal from the needle array.
(MP4)

S2 Movie Electrical stimulation of tubular cardiac constructs. This movie shows the effects of electrical stimulation (2 Hz) in the fabricated tubular cardiac constructs.
(MP4)

## References

[1] Redfield MM. Heart failure—an epidemic of uncertain proportions *N*. *Engl*. *J*. *Med*. 2002; 347 1442–4 10.1056/NEJMe020115 12409548 10.1056/NEJMe020115

[2] Sousa M, Monohan G, Rajagopalan N, Grigorian A, Guglin M. Heart transplantation in cardiac amyloidosis *Heart Fail*. *Rev*. 2017; 22 317–27 10.1007/s10741-017-9601-z 28281017 10.1007/s10741-017-9601-z

[3] Stewart GC, Mayer JE Jr. Heart transplantation in adults with congenital heart disease. *Heart Fail*. *Clin*. 2014;10 207–18 10.1016/j.hfc.2013.09.007 24275305 10.1016/j.hfc.2013.09.007

[4] Stehlik J, Edwards LB, Kucheryavaya AY, Benden C, Christie JD, Dipchand AI et al The Registry of the International Society for Heart and Lung Transplantation: 29th official adult heart transplant report—2012 *J*. *Heart Lung Transplant*. 2012;31 1052–64 10.1016/j.healun.2012.08.002 22975095 10.1016/j.healun.2012.08.002

[5] Miller LW, Guglin M, Rogers J. Cost of ventricular assist devices: can we afford the progress? *Circulation* 2013;127 743–8 10.1161/CIRCULATIONAHA.112.139824 23401115 10.1161/CIRCULATIONAHA.112.139824

[6] Kadakia S, Moore R, Ambur V, Toyoda Y. Current status of the implantable LVAD *Gen*. *Thorac*. *Cardiovasc*. *Surg*. 2016;64 501–8 10.1007/s11748-016-0671-y 27270581 10.1007/s11748-016-0671-y

[7] Langer R, Vacanti J. Advances in tissue engineering *J*. *Pediatr*. *Surg*. 2016; 51 8–12 10.1016/j.jpedsurg.2015.10.022 26711689 10.1016/j.jpedsurg.2015.10.022 PMC4733916

[8] Takahashi K, Tanabe K, Ohnuki M, Narita M, Ichisaka T, Tomoda K, et al Induction of pluripotent stem cells from adult human fibroblasts by defined factors *Cell*. 2007; 131 861–72 10.1016/j.cell.2007.11.019 18035408 10.1016/j.cell.2007.11.019

[9] Okita K, Yamanaka S Induced pluripotent stem cells: opportunities and challenges *Philos*. *Trans*. *R*. *Soc*. *Lond*. *B*. *Biol*. *Sci*. 2011;366 2198–207 10.1098/rstb.2011.0016 21727125 10.1098/rstb.2011.0016 PMC3130417

[10] Tran TH, Wang X, Browne C, Zhang Y, Schinke M, Izumo S, et al Wnt3a-induced mesoderm formation and cardiomyogenesis in human embryonic stem cells *Stem Cells*. 2009; 27 1869–78 10.1002/stem.95 19544447 10.1002/stem.95

[11] Kattman SJ, Witty AD, Gagliardi M, Dubois NC, Niapour M, Hotta A, et al Stage-specific optimization of activin/nodal and BMP signaling promotes cardiac differentiation of mouse and human pluripotent stem cell lines *Cell Stem Cell* 2011;8 228–40 10.1016/j.stem.2010.12.008 21295278 10.1016/j.stem.2010.12.008

[12] Tohyama S, Hattori F, Sano M, Hishiki T, Nagahata Y, Matsuura T, et al Distinct metabolic flow enables large-scale purification of mouse and human pluripotent stem cell-derived cardiomyocytes *Cell Stem Cell*. 2013;12 127–37 10.1016/j.stem.2012.09.013 23168164 10.1016/j.stem.2012.09.013

[13] Godier-Furnémont AF, Tiburcy M, Wagner E, Dewenter M, Lämmle S, El-Armouche A, et al Physiologic force-frequency response in engineered heart muscle by electromechanical stimulation. *Biomaterials* 2015;60 82–91 10.1016/j.biomaterials.2015.03.055 25985155 10.1016/j.biomaterials.2015.03.055 PMC4921199

[14] Tandon N, Taubman A, Cimetta E, Saccenti L, Vunjak-Novakovic G. Portable bioreactor for perfusion and electrical stimulation of engineered cardiac tissue *Conf*. *Proc*. *IEEE Eng*. *Med*. *Biol*. *Soc*. 2013 6219–23 10.1109/EMBC.2013.6610974 24111161 10.1109/EMBC.2013.6610974 PMC4476524

[15] Jawad H, Lyon AR, Harding SE, Ali NN, Boccaccini AR. Myocardial tissue engineering. *Br*. *Med*. *Bull*. 2008; 87 31–47 10.1093/bmb/ldn026 18790825 10.1093/bmb/ldn026

[16] Sakaguchi K, Shimizu T, Okano T. Construction of three-dimensional vascularized cardiac tissue with cell sheet engineering *J*. *Control*. *Release*. 2015;205 83–8 10.1016/j.jconrel.2014.12.016 25523520 10.1016/j.jconrel.2014.12.016

[17] Kikuchi T, Shimizu T, Wada M, Yamato M, Okano T. Automatic fabrication of 3-dimensional tissues using cell sheet manipulator technique *Biomaterials* 2014; 35 2428–35 10.1016/j.biomaterials.2013.12.014 24370007 10.1016/j.biomaterials.2013.12.014

[18] Sawa Y, Miyagawa S, Sakaguchi T, Fujita T, Matsuyama A, Saito A, et al. Tissue engineered myoblast sheets improved cardiac function sufficiently to discontinue LVAS in a patient with DCM: report of a case *Surg*. *Today*. 2012; 42 181–4 10.1007/s00595-011-0106-4 22200756 10.1007/s00595-011-0106-4

[19] Sekine H, Shimizu T, Hobo K, Sekiya S, Yang J, Yamato M, et al. Endothelial cell coculture within tissue-engineered cardiomyocyte sheets enhances neovascularization and improves cardiac function of ischemic hearts. Circulation. 2008 9 30;118(14 Suppl):S145–52 10.1161/CIRCULATIONAHA.107.757286 18824746 10.1161/CIRCULATIONAHA.107.757286

[20] Jakab K, Norotte C, Damon B, Marga F, Neagu A, Besch-Williford CL, et al. Tissue engineering by self-assembly of cells printed into topologically defined structures *Tissue Eng*. *Part A* 2008; 14 413–21 10.1089/tea.2007.0173 18333793 10.1089/tea.2007.0173

[21] Noguchi R, Nakayama K, Itoh M, Kamohara K, Furukawa K, Oyama J, et al. Development of a three-dimensional pre-vascularized scaffold-free contractile cardiac patch for treating heart disease *J*. *Heart Lung Transplant*. 2016; 35 137–45 10.1016/j.healun.2015.06.001 26433566 10.1016/j.healun.2015.06.001

[22] Ong CS, Fukunishi T, Zhang H, Huang CY, Nashed A, Blazeski A et al. Biomaterial-free three-dimensional bioprinting of cardiac tissue using human induced pluripotent stem cell derived cardiomyocytes *Sci*. *Rep*. 2017; 7 4566 10.1038/s41598-017-05018-4 28676704 10.1038/s41598-017-05018-4 PMC5496874

[23] Sekine H, Shimizu T, Yang J, Kobayashi E, Okano T. Pulsatile myocardial tubes fabricated with cell sheet engineering *Circulation* 2006;114 I87–93 10.1161/CIRCULATIONAHA.105.000273 16820651 10.1161/CIRCULATIONAHA.105.000273

[24] Seta H, Matsuura K, Sekine H, Yamazaki K, Shimizu T. Tubular Cardiac Tissues Derived from Human Induced Pluripotent Stem Cells Generate Pulse Pressure In Vivo. Sci Rep. 2017 3 30;7:45499 10.1038/srep45499 28358136 10.1038/srep45499 PMC5371992

[25] Grimm FA, Iwata Y, Sirenko O, Bittner M, Rusyn I. High-content assay multiplexing for toxicity screening in induced pluripotent stem cell-derived cardiomyocytes and hepatocytes *Assay Drug Dev. Technol*. 2015; 13 529–46 26539751 10.1089/adt.2015.659 PMC4652224

[26] Kattman SJ, Koonce CH, Swanson BJ, Anson BD. Stem cells and their derivatives: a renaissance in cardiovascular translational research *J*. *Cardiovasc*. *Transl*. *Res*. 2011; 4 66–72 10.1007/s12265-010-9235-1 21061105 10.1007/s12265-010-9235-1

[27] Itoh M, Nakayama K, Noguchi R, Kamohara K, Furukawa K, Uchihashi K, et al Scaffold-free tubular tissues created by a Bio-3D printer undergo remodeling and endothelialization when implanted in rat aortae *PLoS One*. 2015;10 e0136681 10.1371/journal.pone.0136681 26325298 10.1371/journal.pone.0136681 PMC4556622

[28] Yanagi Y, Nakayama K, Taguchi T, Enosawa S, Tamura T, Yoshimaru K, et al. In vivo and ex vivo methods of growing a liver bud through tissue connection *Sci*. *Rep*. 2017;7 14085 10.1038/s41598-017-14542-2 29074999 10.1038/s41598-017-14542-2 PMC5658340

[29] Beauchamp P, Moritz W, Kelm JM, Ullrich ND, Agarkova I, Anson B, et al. Development and characterization of a scaffold-free 3D spheroid model of induced pluripotent stem cell-derived human cardiomyocytes *Tissue Eng*. *Part C Methods* 2015; 21 852–61 10.1089/ten.TEC.2014.0376 25654582 10.1089/ten.TEC.2014.0376

[30] Stevens KR, Kreutziger KL, Dupras SK, Korte FS, Regnier M, Muskheli V, et al. Physiological function and transplantation of scaffold-free and vascularized human cardiac muscle tissue *Proc*. *Natl*. *Acad*. *Sci*. *U S A*. 2009;106 16568–73 10.1073/pnas.0908381106 19805339 10.1073/pnas.0908381106 PMC2746126

[31] van Marion MH, Bax NA, van Turnhout MC, Mauretti A, van der Schaft DW, Goumans MJ, et al. Behavior of CMPCs in unidirectional constrained and stress-free 3D hydrogels *J*. *Mol*. *Cell*. *Cardiol*. 2015;87 79–91 10.1016/j.yjmcc.2015.08.010 26278995 10.1016/j.yjmcc.2015.08.010

[32] Stoppel WL, Kaplan DL, Black LD 3rd. Electrical and mechanical stimulation of cardiac cells and tissue constructs *Adv*. *Drug Deliv*. *Rev*. 2016;96 135–55 10.1016/j.addr.2015.07.009 26232525 10.1016/j.addr.2015.07.009 PMC4698182

[33] Emmert MY, Hitchcock RW, Hoerstrup SP. Cell therapy, 3D culture systems and tissue engineering for cardiac regeneration *Adv*. *Drug Deliv*. *Rev*. 2014; 69–70 254–69 10.1016/j.addr.2013.12.004 24378579 10.1016/j.addr.2013.12.004

[34] Uchida H, Machida M, Miura T, Kawasaki T, Okazaki T, Sasaki K, et al A xenogeneic-free system generating functional human gut organoids from pluripotent stem cells, *JCI Insight*. 2017, 12;2(1):e86492 10.1172/jci.insight.86492 28097227 10.1172/jci.insight.86492 PMC5214546

[35] Mason JW, Ramseth DJ, Chanter DO, Moon TE, Goodman DB, Mendzelevski B. Electrocardiographic reference ranges derived from 79,743 ambulatory subjects *J*. *Electrocardiol*. 2007; 40 228–34 10.1016/j.jelectrocard.2006.09.003 17276451 10.1016/j.jelectrocard.2006.09.003

[36] Radisic M, Park H, Shing H, Consi T, Schoen FJ, Langer R, et al. Functional assembly of engineered myocardium by electrical stimulation of cardiac myocytes cultured on scaffolds *Proc*. *Natl*. *Acad*. *Sci*. *U S A*. 2004;101 18129–34 10.1073/pnas.0407817101 15604141 10.1073/pnas.0407817101 PMC539727

[37] Amano Y, Nishiguchi A, Matsusaki M, Iseoka H, Miyagawa S, Sawa Y, et al. Development of vascularized iPSC derived 3D-cardiomyocyte tissues by filtration Layer-by-Layer technique and their application for pharmaceutical assays *Acta Biomater*. 2016;33 110–21 10.1016/j.actbio.2016.01.033 26821339 10.1016/j.actbio.2016.01.033

