# Correction: Fabrication of scaffold-free tubular cardiac constructs using a Bio-3D printer

**DOI:** 10.1371/journal.pone.0243244

**Published:** 2020-11-25

**Authors:** Kenichi Arai, Daiki Murata, Ana Raquel Verissimo, Yosuke Mukae, Manabu Itoh, Anna Nakamura, Shigeki Morita, Koichi Nakayama

There is an error in the Competing Interests statement. The correct Competing Interests statement is as follows: Co-author K. Nakayama is a co-founder and shareholder of Cyfuse Biomedical KK. and an inventor/ developer designated on the patent for the Bio-3D printer. Patent title: Method for Production of Three-Dimensional Structure of Cells; patent number: JP4517125. Patent title: Cell Structure Production Device; patent number: JP5896104. The other authors have declared that no competing interests exist. This does not alter the authors’ adherence to PLOS ONE policies on sharing data and materials.
